# Inspecting the “health poverty trap” mechanism: self-reinforcing effect and endogenous force

**DOI:** 10.1186/s12889-024-18464-3

**Published:** 2024-03-28

**Authors:** Liping Fu, Tong Pei, Jiangtao Xu, Jiarui Han, Jie Yang

**Affiliations:** 1 College of Management and Economics, Tianjin University, No.92 Weijin Rd, Tianjin, China; 2 College of Politics and Public Administration, Qinghai Minzu University, Xining, Qinghai Province China; 3 School of Marxism, Hainan University, Haikou, Hainan Province China; 4 School of Public Administration, Hainan University, Haikou, Hainan Province China

**Keywords:** Health poverty trap, Health inequality, Health gradient, Digital literacy, Unconditional quantile regression

## Abstract

**Introduction:**

The term “health poverty trap” describes a vicious cycle in which developing countries or regions become trapped in low levels of health and poverty during the process of modernization. Although significant progress has been made in alleviating poverty in China, there is still a need to further enhance the living conditions of its impoverished population.

**Methods:**

This research utilizes the data of the three national representative panel surveys from 2014 to 2020. The primary objective is to gain a better understanding of the intricate relationship between health and poverty. To examine the self-reinforcing effects of the cumulative cycle between health and poverty, we employ unconditional quantile regression analysis.

**Result:**

The low-income group exhibits lower overall health status compared to the average level. Economic constraints partially hinder the ability of low-income individuals to access healthcare resources, thereby reinforcing the cyclical relationship between health and poverty. Additionally, the unique psychological and behavioral preferences of individuals in health poverty act as indirect factors that further strengthen this cycle. Health poverty individuals can generate endogenous force to escape the “health poverty trap” by enhancing their confidence levels and digital literacy.

**Conclusions:**

The research examines the coexistence of health gradients and economic inequality among Chinese residents. Additionally, the study explores the endogenous force mechanism of escaping the health poverty trap from psychological and behavioral perspectives. This research also offers insights into optimizing government poverty alleviation programs to effectively address this issue.

## Background

The relationship between health and economic growth has long been a subject of keen interest for researchers across various disciplines, including economics, sociology, and demography. In the context of global economic development, considerable disparities exist in income levels and health outcomes among nations [1]. Developed countries with higher incomes tend to enjoy better overall health, exhibiting a significant positive correlation between income and health [2]. In contrast, developing countries often find themselves trapped in a cycle of low income, low consumption, and poor health [3]. Despite the diverse levels of progress achieved in economic growth, developing nations have faced significant obstacles in effectively mitigating poverty. The issue of widespread and enduring poverty remains a pressing and formidable challenge for these countries [4, 5].

The 2019 Nobel Prize in Economics was awarded to three development economists who proposed experimental approaches to alleviating global poverty, prompting people to reflect on practical solutions to poverty [6]. According to Nobel laureates Abhijit V. Banerjee and others, even if sufficient resources are provided to people experiencing poverty, it does not guarantee the avoidance of a “poverty trap” [7]. Their empirical research examined 18 impoverished countries and regions, concluding that people remain poor because they do not effectively allocate resources to aspects that promote their sustained and positive development, such as health and nutrition. In other words, the inability of the impoverished population to escape from their current circumstances contributes to worsening their health conditions. This reciprocal cause-and-effect relationship results in a vicious cycle of poverty, deepening the plight of individuals trapped within it, thereby perpetuating a long-term state of low-level equilibrium [8, 9]. The phenomenon known as the “health poverty trap” emerges from the interplay between deteriorating health resulting from poverty and the persistent inability to escape poverty [10]. This state is characterized by the mutual reinforcement of poverty-induced health decline and the ongoing cycle of poverty, creating a situation where individuals or communities are trapped in a cycle of poor health and persistent poverty.

From 1978 to the end of 2018, the rural poverty population in China witnessed a significant decline from 770 million to 16.6 million individuals, resulting in a poverty incidence rate of 1.7%. As of 2020, absolute poverty has been largely eradicated according to China’s current poverty standards. Consequently, the focus of poverty alleviation efforts has shifted towards addressing and mitigating multidimensional relative poverty stemming from imbalances and inadequacies in development [11]. Throughout the course of poverty alleviation, it has become increasingly evident that health risks extend beyond the realm of illness. They also have adverse effects on labor and economic income. The interconnection between health and poverty is intricately woven. From a household perspective, the health status of family members directly impacts the quantity and quality of household labor. Poor health not only jeopardizes income stability but also diminishes the overall living standards of the household, resulting in a state of poverty [12]. Consequently, phenomena such as “poverty due to illness” and “falling back into poverty due to illness” emerge. Understanding the causal relationship between health and poverty, as well as comprehending the unique psychological and health behavioral preferences of individuals trapped in the health poverty cycle, is crucial for addressing this issue [13].

## Literature review

The theory of the poverty trap in developing countries has its roots in the early days of the emergence of development economics during the 1950s. Notable theories in this realm include Nurkse’s theory of the “vicious circle of poverty,” Nelson’s theory of the “low-level equilibrium trap,” and Myrdal’s theory of “circular cumulative causation” [11, 14, 15]. However, in the late 1960s, with the resurgence of neoclassical economics in the field of development economics, the development of poverty trap theory experienced a gradual deceleration [16].From the 1980s onward, research began to focus on the mechanisms through which poverty depletes individuals’ psychological resources. This resulted in the emergence of the psychological trap theory of poverty, approached from a behavioral economics perspective [17]. Moving into the 21st century, a group of economists capitalized on advancements in new growth theory, information economics, new institutional economics, and econometric techniques to advance the study of poverty traps. This involved shifting the analysis of poverty traps from a macro-level to a micro-level and from theoretical explanations to empirical investigations [18].

Existing literature has extensively researched the issue of poverty traps and can be categorized into the following two areas: firstly, economists have attempted to explore the mechanisms underlying the formation of poverty traps, which are the underlying factors that lead to persistent poverty [19, 20]. In traditional poverty trap theories, sufficient material capital and investment are key to escaping poverty. However, contemporary studies on poverty traps focus on population growth and material capital and consider the impact of natural resources, geographical environment, cultural education, political institutions, crime, and disease and disasters [21]. Secondly, researchers have proposed diverse poverty alleviation measures from different perspectives. Escaping the poverty trap requires a significant increase in investment in human capital to drive sustained and stable economic growth [22]. Owen and Barclay suggest a poverty reduction strategy from an institutional standpoint [23]. Specifically, the focus is on effectively implementing specific policies at the micro-level, while institutional innovation is pursued at the macro-level. The choice of poverty reduction strategies should be aligned with the level of economic development [24]. In regions with lower levels of economic development, universally applicable poverty reduction policies are considered more suitable. However, targeted poverty reduction strategies become necessary as the economy progresses to help individuals accumulate greater human capital through precise policies [25].

The issue of health and poverty has attracted significant academic attention. From a resource endowment perspective, health is considered a natural capital accompanying individuals throughout their life cycle [26, 27]. Research has shown a gradient in health outcomes based on differences in economic income [28, 29]. Specifically, individuals with lower economic and social status face notable disadvantages in terms of health compared to those with higher economic and social status, resulting in health inequalities. When health inequalities, income, and poverty intertwine, there is a significant risk of being trapped, or even locked, in a “health poverty trap“ [30, 31]. This trap can be described as a sequence of events: health inequalities leading to health impairment among low-income populations, which triggers the onset of diseases. Subsequently, opportunities are lost, capabilities are deprived, and economic hardship intensifies, causing a decline in earning capacity. Ultimately, individuals find themselves trapped in a cycle of poverty they cannot escape. Moreover, it is worth noting that the impact of income disparity on health can have a delayed effect [32]. Specifically, when there is a significant income gap, it can have detrimental effects on health. Despite extensive research on the causal relationship between health and poverty, there remains a limited understanding of the challenges associated with breaking free from the cycle of low-level accumulation of health and poverty. The self-reinforcing effects and endogenous forces of poverty alleviation are not well explored, particularly in China, where empirical evidence is scarce. China’s poverty alleviation efforts have indeed achieved tremendous success [33]. However, the current challenge lies in how to further improve the living standards of the impoverished population. This is not only a theoretical issue but also an urgent reality.

In numerous studies within the field of welfare economics, it is widely acknowledged that the health status of a population is associated with their income [34, 35]. However, this correlation is recognized as imperfect. Angus S. Deaton, recipient of the 2015 Nobel Prize in Economics, contends that income inequality, in and of itself, is not inherently harmful to health. Instead, he posits that as income inequality widens, the positive impact of higher personal incomes on health becomes more pronounced [34]. Concurrently, low-income groups contend with a relative scarcity of health resources, particularly when engaged in physically demanding occupations. Consequently, while the affluent experience better health, the health disparity between low-income groups and their wealthier counterparts expands. This paper advances the hypothesis of a direct self-reinforcing effect within the cyclic relationship of health and poverty. Compared to previous research, this research innovates using the latest nationwide representative data to assess the health gradient among Chinese residents and examine the coexistence of health inequality and economic inequality. This study employs a descriptive analysis approach to examine health indicators across varying economic income levels. These health indicators manifest in both the subjective realm, as observed in the health gradient of Self-Rated Health (SRH) indicators, and the objective domain, as indicated by the total cost of medical treatment incurred in the previous year.

Furthermore, the concept of an indirect self-reinforcing effect pertains to specific psychological and behavioral tendencies among economic individuals that intensify the self-perpetuating cycle of health poverty, impeding endogenous motivation to escape the health poverty trap. Within the framework of behavioral economics, studies have transitioned from examining surface causality to delving into the internal micro-behavior that underpins the relationship between health and poverty [36, 37]. Essentially, the low-level equilibrium between health and poverty is posited to be self-reinforcing through individuals’ psychological and behavioral inclinations. This process involves the endogenous motivation required to break free from the poverty trap. In this research, unconditional quantile regression analysis is employed to examine the self-reinforcing effects of the cumulative cycle between health and poverty. Additionally, the research explores the endogenous mechanisms involved in breaking free from the health poverty trap, taking into account psychological and behavioral perspectives. It aims to uncover the essence of health poverty and clarify the directions for optimizing government poverty alleviation programs.

## Methods

Unconditional Quantile Regression (UQR) comprehensively analyzes explanatory variables’ influence on the dependent variable’s unconditional distribution [39]. UQR serves as a complementary method to Conditional Quantile Regression (CQR). Within econometrics, UQR is recognized as a primary technique for uncovering heterogeneity and identifying differential effects across different quantiles [38]. By estimating the results of the independent variables at different quantiles, UQR can identify the differences in impact across various quantiles within the same sample. In this research, the adoption of UQR methods allows for the detection of the indirect self-reinforcement effects between income and variables related to psychological and behavioral preferences, as well as the heterogeneity across the income distribution and urban-rural divide. The Recentered Influence Function (RIF) is commonly defined as follows:

1 $$\eqalign{& RIF\left( {y;\nu,{F_Y}} \right) = \cr & \nu ({F_Y}) + IF\left( {y;\nu,{F_Y}} \right) = \cr & \nu ({F_Y}) + \partial \nu [(1 - t){F_Y} + t{\Delta _y}]/\partial t{|_{t = 0}} \cr} $$

In this context, $$ y $$represents the dependent variable, $$ {F}_{y }$$represents the cumulative contribution of $$ y$$ and $$ v\left({F}_{y}\right)$$represents a real-valued functional. $$ {\varDelta }_{y} $$represents the indicator variable that takes the value of 1 at $$ y$$ and 0 elsewhere, indicating the probability measure. In the case of quantiles, the RIF is typically expressed as:

2 $$RIF\left( {y{\text{;}}{q_\tau }} \right)={q_\tau }+\frac{{\tau - {\text{1\{ }}y \leqslant {q_\tau }{\text{\} }}}}{{{f_{\text{Y}}}({q_\tau })}}$$

where ${q}_{\tau }$ represents the value of the dependent variable at that particular quantile $\tau$. $1\left\{y\le {q}_{\tau }\right\}$corresponds to an indicator function that takes the value of 0 or 1, distinguishing whether the value is an outcome variable. ${f}_{Y}\left(\bullet \right)$ refers to the estimated density function of the dependent variable obtained from the sample.

3 $$p[RIF({y_{it}}{\text{;}}{q_\tau })=\tau /{f_{\text{Y}}}({q_\tau })|{X_{it}},{c_i}]=X_{{it}}^{\prime }[{f_{\text{Y}}}({q_\tau })\beta ]+{c_i}$$

${X}_{it}$indicates all treatment and control variables,${c}_{i}$represents individual fixed effects.

4 $$\eqalign{& p[RIF({y_{it}};{q_\tau }) = \cr & \tau /{f_{\rm{Y}}}({q_\tau })|{X_{it}},{c_i}]/{f_{\rm{Y}}}({q_\tau }) \cr & + {q_\tau } + (\tau - 1)/{f_{\rm{Y}}}({q_\tau }) \cr & = X_{it}^\prime \beta + {c_i} \cr} $$

Since ${c}_{i}$captures all constant terms, Eq. (3) can be simplified to the following form:

## Data

The primary data source for this research is the China Family Panel Studies (CFPS) microdata. The data collection process utilized a stratified multi-stage random sampling method covering 25 provinces in China. CFPS is a nationally representative and high-quality database widely employed in various social science disciplines [39]. For this analysis, the authors utilized CFPS panel data from 2016, 2018, and 2020. To ensure data accuracy and completeness, the authors first excluded underage respondents and individuals with responses such as “don’t know,” “refused to answer,” “not applicable,” or missing values for each indicator. After this exclusion, the final sample consisted of 37,219 respondents in Table 1, with 18,239 residing in urban areas and 18,980 in rural areas.

**Table 1:** Descriptive statistics

Variable	Definition	Full Samples Obs = 37,219		City Residents Obs = 18,239		Rural Residents Obs = 18,980	
		Mean	S.D.	Mean	S.D.	Mean	S.D.
**Dependent Variables**							
Incidence of Health Poverty	1 = poor health and poverty occur simultaneously; 0 = otherwise	0.05	0.22	0.02	0.14	0.08	0.28
Health Status	1–5 represents self-rated health from very healthy to very unhealthy	2.90	1.16	2.85	1.13	2.95	1.18
Income	Annual personal economic income (RMB)	29635.73	65609.13	33287.79	39956.04	26126.25	82957.77
**Explanatory Variables**							
Digital Literacy	1–4 represents digital literacy levels from weak to strong	2.65	1.16	2.79	1.11	2.51	1.19
Confidence	1–5 represents confidence level from very unconfident to very confident	4.07	0.96	4.11	0.92	4.03	1.00
Exercise Frequency	2 = often; 1 = sometimes; 0 = never	0.80	0.95	0.88	0.96	0.71	0.94
Medical Decisions	1–5 represents selection of medical institution level from low to high	3.20	1.61	3.32	1.63	3.08	1.58
**Control Variables**							
Age	Years	44.59	15.88	43.22	15.18	45.91	16.41
Gender	1 = male; 0 = female	0.56	0.50	0.56	0.50	0.57	0.50
Marriage	1 = married; 0 = otherwise	0.79	0.40	0.79	0.41	0.80	0.40
Policital Status	1 = With a political identity; 0 = No political identity	0.04	0.19	0.02	0.16	0.05	0.22
Education	Years of schooling	7.38	5.05	8.85	4.60	5.97	5.05
Family Size	The number of members in the household	3.98	2.06	3.83	2.01	4.12	2.10
Medical convenience	1–5 represents the transportation convenience of seeking medical treatment from not convenient to convenient	3.26	0.96	3.47	0.90	3.05	0.98
Medical Service Level	1–5 represents medical service capacity from low to high	3.30	0.93	3.46	0.88	3.14	0.95

The first dependent variable is the incidence of health poverty, represented as a dummy variable where the occurrence is denoted as one and non-occurrence as zero. The coexistence of poor health and economic poverty serves as two key indicators to determine the prevalence of health poverty. In existing independent measurement studies, the interpretation of the concept of health poverty is mostly based on a single dimension or indicator of self-assessment of health status and economic poverty [40, 41]. For example, Pascual-Sae used the traditional poverty measurement method (Foster-Greer-Thorbecke, FGT) to study the dynamic changes in health poverty in Spain, an measured health poverty through self-assessment of health [42]. At present, there is little theoretical research on health poverty, mostly relying on capacity poverty theory and multidimensional poverty theory. Regarding economic indicators, the CFPS survey questionnaire provides information on individuals’ annual total financial income. Regarding the selection of the poverty line, this study utilizes the local minimum living standard from the first quarter of the survey year [43]. For instance, in Guangdong, the minimum living allowances for urban and rural residents were 6,180 yuan and 4,513 yuan, respectively, in 2016. By 2020, these values had increased to 10,008 yuan and 7,998 yuan, respectively, in urban and rural areas of Guangdong Province. Regarding health indicators, individuals with self-rated health (SRH) scores of 5 points in the CFPS are considered unhealthy. SRH scores are consistent with objective health status and can be used as a global measure of the health status of the general population [44–46]. Considering economic and health indicators, individuals with SRH scores of 5 and a personal income below the local minimum living standard are defined as experiencing health poverty. Based on calculations, 1958 individuals in the sample experience health poverty, resulting in an approximate health poverty occurrence rate of 5%. Among them, the urban health poverty occurrence rate is 2%, while the rural health poverty occurrence rate is 8%. This shows that there is a clear urban-rural dichotomy in the incidence of health poverty among Chinese residents, with rural residents having a higher incidence of health poverty than urban residents.

Taking into consideration that disparities in health behaviors play a crucial role in contributing to health inequality, this research has specifically chosen four pertinent variables associated with individual psychological factors and preferences in health behavior. Specifically, this article has focused on a variable related to health psychology. Previous research has shown that confidence has a positive impact on SRH [47]. The assessment of respondents’ confidence in their future provides insights into their subjective belief in their capacity to overcome current circumstances. Individuals with higher levels of confidence generally demonstrate a more optimistic perspective on life and possess greater resilience in the face of challenges. In the CFPS database, a grading system ranging from 1 to 5 is employed to quantify confidence levels, with 1 indicating very low confidence and 5 representing very high confidence.

Concerning variables related to health behavior, the CFPS questionnaire included three relevant indicators. One of the related variables about health behavior is the frequency of exercise, which mainly refers to physical activity in and out of the house for strong physical fitness, pleasure, and so on, and does not include cycling and walking for the sole purpose of going to work. The frequency is divided into three levels, no exercise = 0, exercise less than once a week = 1, and exercise not less than one time per week = 2.

Digital literacy is one of the variables associated with health behavior, and it is measured in four dimensions: digital use, digital life, digital learning, and digital information [48–51]. Digital use is assessed through the question in the questionnaire: “Do respondents use mobile networks?” Then digital life is characterized by the continuous variable of daily internet usage duration reported by the respondents. Specifically speaking, a longer internet usage duration implies a higher frequency of digital technology usage. For the digital learning measure, a value of one is assigned to taking various courses or attending online training; otherwise, it is zero. This research also includes the dimension of digital information, which categorizes the significance of obtaining information via the Internet into five levels from 1 to 5. A higher level reflects a larger reliance on Internet-based information retrieval. Finally, this investigation uses a two-step clustering analysis to categorize the 37,367 observed sample values into four levels. A higher level indicates that this population utilizes digital technology more frequently and has a higher level of digital literacy.

The indicator of medical decisions refers to the type of institution that people generally select when they become unwell. The literature demonstrates that, when seeking medical treatment, residents tend to go directly to higher-level hospitals, rather than to primary medical institutions [52, 53]. China issued “Guiding Opinions on Promoting the Construction of the Hierarchical Diagnosis and Treatment System” in 2015 [54]. With the continuous advancement of the Hierarchical Diagnosis and Treatment System, the level of services at primary medical institutions has improved significantly. Likewise, patients are rationally assigned to various levels of medical institutions based on their condition. The assignment of values to this variable is based on the level of the medical institution, clinic = 1, community health service station/village health room = 2, community health services center/town health institution = 3, specialized hospital = 4, general hospital = 5. As indicated in Table 1, it can be observed that rural residents generally prefer lower levels of care compared to urban residents.

This research provides a descriptive analysis of other control variables. Drawing upon existing research literature and considering heterogeneity issues, the empirical analysis controls for other potential factors that may influence individual health, including education level, age, gender, marital status, family size, medical convenience, medical service level and rural-urban location [55, 56]. Age, education level, and family size are treated as continuous or ordinal variables, while the remaining variables are categorical. A fixed-effects model with provincial variables is employed to address the economic inequality resulting from provincial differences. This research applies natural logarithm transformations to the income and education variables to address heteroscedasticity concerns. Additionally, the age variable is squared and divided by 100.

## Results analysis

The low-level cumulative cycle between health and poverty includes direct and indirect self-reinforcing effects [57]. The former refers to the direct reinforcing relationship between health and poverty. According to the theory of health gradients, health tends to vary with changes in economic development [58]. Specifically, individuals with higher levels of economic income have access to more health resources. Wealthier individuals in the same geographical area tend to be healthier than those living in poverty. Conversely, individuals facing economic pressures and inadequate access to health resources may experience health deterioration issues. To examine this, the research first employs the theory of health gradients to test whether health status follows a gradient distribution with changes in income. If the health status of the low-income group is also poor, it suggests the presence of a direct self-reinforcing effect between health and poverty. In this research, the definition of the low-income group is based on the reference from the “China Statistical Yearbook,” which defines it as the bottom 20% of income earners. The high-income group refers to the top 20% of income earners, while the remaining individuals are collectively referred to as the middle-income group [59]. Simultaneously, the middle-income group will be divided into upper-middle-income and lower-middle-income groups.

Furthermore, this research utilizes a UQR method to explore the relationship between health status and economic income specifically among the low-income population. The purpose is to investigate whether there is stratification of health across different income groups. This provides further evidence for the self-reinforcing relationship between health and poverty. Indirect self-reinforcing effects refer to the self-perpetuating cycle of health poverty intensified by individuals’ psychological and behavioral preferences, which inhibit the intrinsic motivation to escape the “health poverty trap”. To address this, the research includes four core explanatory variables in the regression analysis: digital literacy, medical decisions, confidence in the future, and frequency of physical exercise. This is done to examine whether there are constraint effects in place.

### Direct self-reinforcement Effect

Based on the latest CFPS cross-section data, it is observed that the health level of Chinese residents significantly improves with increasing economic income. Additionally, there are notable differences in health levels among different economic income groups in 2020. Table 2 shows the proportion of individuals with SRH scores of 1 and 2 in each income group. In the overall sample, the percentage of individuals with good health within the low-income group is only 24.26%. More specifically, rural residents account for 22.60% of this group, while urban residents account for 25.00%. In rural areas, there is a significant disparity in health status among different income groups. However, among urban residents, there is relatively little difference in health status between the middle-income and high-income groups. Regarding inter-group health gradient differences, the health gradient between the 20th and 80th percentiles of individual economic income is 9.85%, with the health gradient for urban residents being 6.35% and for rural residents being 17.42%. In Table 3, objective indicators of physical condition are also provided, specifically, the median total medical expenses in the past 12 months for each income group. The health status of low-income groups is significantly lower than that of both middle-income and high-income groups. Specifically, within the rural population, the median total medical expense in the high-income group is 795 yuan lower than in the low-income group. Similarly, in the urban population, the median total medical expense in the middle-income group is 200 yuan lower than that in the low-income group. Additionally, there is some fluctuation in the median total medical expense for urban high-income individuals.

**Table 2:** Descriptive analysis of economic income level and health gradient

Variable	Income (< 20%)	Income (20% ∼50%)	Income (50% ~ 80%)	Income (> 80%)	Health Gradient
Rural Healthy Residents	22.60%	30.20%	36.66%	40.02%	**17.42%**
the Median of Annual Total Medical Expenses (CNY)	800	200	60	5	**795**
Obs	(1177)	(1765)	(1765)	(1177)	-
City Healthy Residents	25.00%	28.58%	29.56%	31.35%	**6.35%**
the Median of Annual Total Medical Expenses (CNY)	300	100	100	150	**200**
Obs	(1576)	(2365)	(2365)	(1576)	-
Full Sample Healthy Residents	24.26%	30.14%	32.15%	34.11%	**9.85%**
the Median of Annual Total Medical Expenses (CNY)	500	100	100	100	**400**
Obs	(2753)	(4130)	(4130)	(2753)	-

The analysis of the health gradient reveals a notable urban-rural disparity in the health status of individuals, with a substantial gap observed between the health of the low-income group in rural areas and the middle-income and high-income groups. First, low-income groups in rural areas often engage in manual labor, which can lead to poorer health outcomes. In addition, rural residents face relatively limited access to health resources, and thus their health status is more likely to deteriorate compared to urban residents. From a health gradient perspective, health inequality among Chinese residents coexists with income inequality, particularly in rural areas. While income inequality itself may not directly harm health, the widening income gap underscores the increasing importance of individual income in improving health outcomes [60]. It becomes increasingly evident that the low-income group faces greater constraints in accessing health resources, which further reinforces the cyclical relationship between health and poverty at a lower socioeconomic level.

**Fig. 1:**
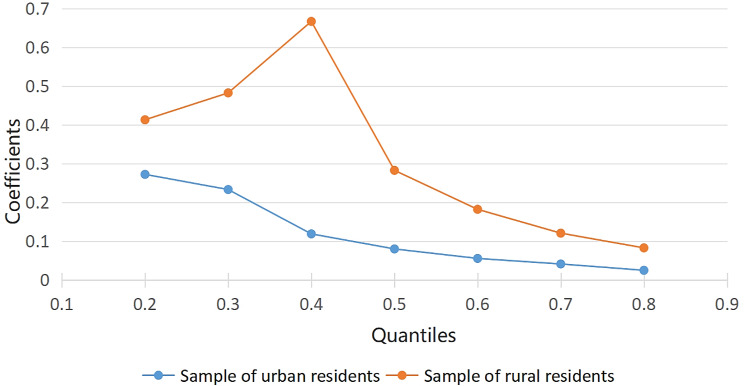
Quantile regression results based on urban-rural heterogeneity

To further examine the self-reinforcing effect of health and poverty in the low-level cycle, this research employs a URQ model to investigate the impact of health on economic income at different income quantiles of panel data from 2016 to 2020 (as shown in Table 3). In general, health status is significantly associated with economic income at different quantiles, and there are significant variations in the coefficient changes. In rural areas, the impact of health status is most significant for the low and medium-income group, with an estimated coefficient of 0.6667. The coefficients at the 20th and 80th percentiles are 0.4130 and 0.826, respectively, exhibiting an inverted U-shaped distribution (as shown in Fig. 1). The results indicate that the impact of improving health on increasing economic income is less pronounced for high-income individuals, while it is more significant for those in the middle and lower-income groups. This suggests that lower-income rural populations can achieve a virtuous cycle of health improvement and income growth.

**Table 3:** Quantile regression results based on urban-rural heterogeneity

Variable	Full Sample	Urban_q20	Urban_q30	Urban_q40	Urban_q50	Urban_q60	Urban_q70	Urban_q80
Health Status	-0.0998^***^ (0.0187)	-0.2722^***^ (0.0347)	-0.2331^***^ (0.0290)	-0.1187^***^ (0.0153)	-0.0799^***^ (0.0140)	-0.0554^***^ (0.0112)	-0.0411^***^ (0.0077)	-0.0248^***^ (0.0086)
Other control variables	Yes	Yes	Yes	Yes	Yes	Yes	Yes	Yes
Province	-	Yes	Yes	Yes	Yes	Yes	Yes	Yes
Obs	18,234	18,234	18,234	18,234	18,234	18,234	18,234	18,234
**Variable**	**Full Sample**	**Rural_q20**	**Rural_q30**	**Rural_q40**	**Rural_q50**	**Rural_q60**	**Rural_q70**	**Rural_q80**
Health Status	-0.2089^***^ (0.0227)	-0.4130^**^ (0.1448)	-0.4824^***^ (0.0417)	-0.6667^***^ (0.0442)	-0.2826^***^ (0.0172)	-0.1820^***^ (0.0094)	-0.1206^***^ (0.0073)	-0.0826^***^ (0.0051)
Other control variables	Yes	Yes	Yes	Yes	Yes	Yes	Yes	Yes
Province	-	Yes	Yes	Yes	Yes	Yes	Yes	Yes
Obs	18,947	18,947	18,947	18,947	18,947	18,947	18,947	18,947

*Note* * *p* < 0.1, ** *p* < 0.05, *** *p* < 0.01, corresponding robust standard error in parentheses

For urban residents, the coefficients at the 20th, 50th, and 80th percentiles are 0.2722, 0.0799, and 0.0248, respectively. The evidence suggests that the urban low-income group has a higher probability of increasing their income by improving their health status compared to other groups. This implies a greater likelihood for urban low-income populations to break free from the “health poverty trap,” which may be attributed to the availability of better employment opportunities in urban areas. Furthermore, the impact coefficients of health conditions on middle-income and high-income groups are relatively smaller. This indicates that there is a lower probability for high-income groups to further increase their economic income by improving health conditions in urban settings. In summary, a direct self-reinforcing effect exists between health and poverty specifically within the low-income population as a whole.

### Indirect self-reinforcing Effect

Based on the benchmark regression, four explanatory variables related to psychological and behavioral preferences were added to investigate the indirect self-reinforcing effects of health poverty. From Table 4, it can be observed that digital literacy is positively and significantly correlated with individuals’ economic income. This implies that individuals with access to internet information sources are likely to possess greater health knowledge, emphasize disease prevention and early detection, and positively impact their economic income. However, the other variables show varying results. The coefficients for selecting healthcare institutions show significant differences, with high-income and middle-income populations showing a stronger inclination toward general medical institutions. Conversely, economic constraints have a certain degree of influence on the healthcare seeking decisions of the low-income population, causing them to favor lower-tier medical institutions.

**Table 4:** Results of the quantile regression based on income heterogeneity

Variable	Urban_q20	Rural_q20	Urban_q50	Rural_q50	Urban_q80	Rural_q80
Health Status	-0.2605^***^ (0.0334)	-0.2619^***^ (0.0139)	-0.0751^***^ (0.0144)	-0.2729^***^ (0.0162)	-0.0197^**^ (0.0084)	-0.0757^***^ (0.0045)
Digital Literacy	0.3469^***^ (0.0573)	0.3328^***^ (0.0125)	0.1984^***^ (0.0102)	0.2929^***^ (0.0176)	0.1544^***^ (0.0140)	0.1746^***^ (0.0201)
Medical Decisions	-0.0567^***^ (0.0149)	-0.0319^***^ (0.0108)	0.0407^***^ (0.0058)	0.0767^***^ (0.0145)	0.0621^***^ (0.0072)	0.0622^***^ (0.0076)
Confidence	-0.0143 (0.0298)	-0.0522^**^ (0.0218)	0.0057 (0.0140)	-0.0589^**^ (0.0219)	0.0161 (0.0099)	-0.0091 (0.0106)
Exercise Frequency	-0.0316 (0.0303)	-0.1281 (0.0284)	-0.0122 (0.0114)	-0.1611^***^ (0.0156)	0.0467^***^ (0.0086)	0.0831^**^ (0.0091)
Other control variables	Yes	Yes	Yes	Yes	Yes	Yes
Province	Yes	Yes	Yes	Yes	Yes	Yes
Obs	18,234	18,974	18,234	18,974	18,234	18,974
R²	0.1522	0.1022	0.1869	0.1232	0.1310	0.1247

*Note* * *p* < 0.1, ** *p* < 0.05, *** *p* < 0.01, corresponding robust standard error in parentheses

The variable of confidence does not have a significant impact on economic income. However, an interesting observation can be made from the coefficients: the influence of confidence levels on economic income is negative for rural population. At the same time, it is positive for urban middle and high-income individuals. One possible explanation for this phenomenon is that rural residents, if they have confidence in the future, maybe more content with their current situation and may not prioritize pursuing higher economic income. On the other hand, the urban residents, who have confidence in the future, may set higher aspirations for their lives, leading to higher expectations for increasing their economic income. Regarding physical exercise, there exists a positive correlation between the frequency of physical activity and economic income among high-income groups. This correlation may stem from the busy lifestyles and work commitments prevalent in these groups, resulting in limited opportunities for regular exercise. Engaging in appropriate physical activity not only enhances physical well-being but also positively influences economic income. The coefficients for selecting healthcare institution types show significant differences, with middle and high-income individuals tending to prefer general medical facilities. In the case of low-income groups, the choice of healthcare institution for seeking medical treatment is more likely to be constrained by economic income, with they leaning towards selecting lower-tier medical institutions. When considering other control variables, factors such as advanced age, unstable marital status, large family size, low medical level and inconvenient medical treatment are commonly observed among individuals living in poverty. These factors further diminish their motivation and confidence to ameliorate their circumstances.

### Endogeneity Analysis

Given that endogeneity may arise due to unobservable factors affecting both health and income status, as well as the possibility of mutual causation between health and income, this study employs instrumental variables to address potential estimation bias. Drawing on the concepts proposed by Grossman regarding human capital, health is considered as a form of capital that depreciates with age, with greater health capital typically implying higher productivity and thus higher income [61]. Moreover, Grossman conceptualizes health as a function of individual behavior, introducing the concept of a health production function. To overcome potential endogeneity issues, this study selects smoking, drinking, and insomnia-related indicators as instrumental variables reflecting health status, as evidenced by previous research [62–64]. The instrumental variable estimation results, presented in Table 5, include the baseline regression in column (1) and the 2SLS regression results incorporating control variables in columns (2) and (3). Based on panel data from 25 provinces spanning 2016–2020, the final sample size is 36,101.

**Table 5:** Results of IV (2SLS) estimation

Variable	(1)	(2)	(3)
Health Status	-0.1617^***^ (0.1452)		-0.5589^***^ (0.1488)
Confidence	-0.1609 (0.0173)		-0.0729^**^ (0.0320)
Digital Literacy	0.1991^***^ (0.0163)		0.1808^***^ (0.0186)
Medical Decisions	0.0494^***^ (0.0104)		0.0631^***^ (0.0123)
Exercise Frequency	0.0867^***^ (0.0176)		0.0832^***^ (0.0178)
IV: Smoking		-0.0787^***^ (0.1452)	
IV: Drinking		-0.0643^**^ (0.0273)	
IV: Insomnia		0.2141^***^ (0.0209)	
Other control variables	Yes	Yes	Yes
Province	Yes	Yes	Yes
Time	Yes	Yes	Yes
Weak identification test	-	F Value = 55.4365 > 10 DWH(P Value)= 0.000	

*Note* * *p* < 0.1, ** *p* < 0.05, *** *p* < 0.01

The results in Table 5 indicate that the first-stage F-statistics for instrumental variable regression are all greater than 0, and the Durbin-Wu-Hausman endogeneity tests are significant at the 1% level, suggesting that the selected instrumental variables have strong explanatory power for endogenous variables, thereby confirming the presence of endogeneity issues in the model. Therefore, smoking, drinking, and insomnia can be considered as endogenous explanatory variables for health. Additionally, it is noteworthy that the estimated coefficients for the effects of individual health behaviors (smoking, drinking, insomnia) obtained through instrumental variable regression are significantly higher than those from the baseline regression. This difference suggests that smoking, drinking, and insomnia are detrimental to physical health. Ignoring endogeneity issues would lead to an overestimation of the negative effects of individual health behaviors on self-rated health.

### Endogenous forces to escape the “health poverty trap”

This research places significant emphasis on examining the endogenous force behind escaping the “health poverty trap.” Previous analyses have revealed that individuals belonging to different income groups demonstrate distinct psychological cognitions and behavioral preferences when caught in the cycle of health poverty [65–67]. In other words, these psychological and behavioral factors may act as internal forces that contribute to escaping the health poverty trap under specific circumstances. This study classifies these factors into two dimensions: the influence of confidence levels on individuals’ mindsets and behaviors in escaping poverty (referred to as the confidence motive effect), and the role of behaviors such as exercise, healthcare preferences, and utilization of digital information as crucial internal forces for achieving poverty escape (referred to as the behavioral preference effect). The dependent variable, “incidence of health poverty,” is represented as a binary dummy variable. To investigate the presence of endogenous force in escaping health poverty, this study employs the Probit model.

The findings presented in Table 6 reveal significant effects of each independent variable on the occurrence of health poverty at various levels. Specifically, individuals who have confidence in the future and possess digital literacy exhibit a decreased likelihood of experiencing health poverty. However, the variables related to healthcare decision-making and exercise yield unexpected results, which may be attributed to the influence of national policies. Notably, individuals facing health poverty are more inclined to choose general and specialized hospitals when seeking medical care, a pattern that can be attributed to the extensive coverage provided by China’s medical insurance system. Since 2018, the basic medical insurance coverage rate in China has remained consistently high, surpassing 95% [68]. The presence of a comprehensive medical insurance system has had a significant impact on individuals’ health-seeking behavior by reducing out-of-pocket medical expenses and effectively mitigating the risk of health poverty. Regarding the variable of exercise, it is important to highlight that individuals in the health-poverty group also exhibit a greater inclination towards regular physical exercise. This trend can be directly linked to the national strategy of promoting nationwide fitness and building a healthier China. The introduction of the “Healthy China 2030 Planning Outline” has guided individuals towards adopting a lifestyle that includes regular physical exercise through health education initiatives [69]. To support this objective, local authorities have taken proactive measures to improve exercise environments by establishing fitness venues and installing exercise equipment and facilities. These efforts contribute to the creation of an environment that fosters and encourages a healthier lifestyle for all residents.

**Table 6:** Analysis of endogenous force mechanism

Variable	Full Samples	City Residents	Rural Residents
Confidence	-0.1838^***^ (0.0119)	-0.1688^***^ (0.0237)	-0.1922^***^ (0.0143)
Digital Literacy	-0.1846^***^ (0.0125)	-0.1252^***^ (0.0369)	-0.2325^***^ (0.0153)
Medical Decisions	0.0424^***^ (0.0086)	-0.0011 (0.0153)	0.0878^***^ (0.0108)
Exercise Frequency	-0.0803^***^ (0.0144)	-0.0747^***^ (0.0254)	-0.0498^***^ (0.0179)
Other Control Variables	Yes	Yes	Yes
Pseudo R²	0.2464	0.1542	0.2644
Obs	36,101	17,630	18,471

*Note* * *p* < 0.1, ** *p* < 0.05, *** *p* < 0.01, corresponding robust standard error in parentheses

Based on the aforementioned findings, it can be inferred that the confidence motive effect and the behavioral preference effect play a significant role in escaping the “health poverty trap.” These effects transform the self-reinforcing elements of the low-level cycle of health poverty into endogenous forces for poverty alleviation. One of the fundamental aspects in breaking free from the health poverty trap is the pursuit of economic equality. By addressing economic inequalities, individuals in poverty can overcome the limitations of limited access to healthcare resources. When economic equality is achieved, individuals can fully benefit from advancements in medical technology and digital information, leading to improved health outcomes and overall well-being. Furthermore, it is essential to develop assistance policies that are demand-driven and tailored to the specific psychological cognition and health behaviors of individuals in poverty. Such policies should take into account the unique circumstances and needs of individuals, ensuring that interventions are effective in addressing the root causes of health poverty. In conclusion, this research highlights the importance of considering endogeneity and emphasizes the significance of economic equality and tailored assistance policies in enabling individuals to escape the cycle of health poverty.

## Conclusions

This research provides evidence that health inequality and economic income inequality coexist among the Chinese population. The low-income group exhibits lower overall health status compared to the average level. Economic constraints partially hinder the ability of low-income individuals to access healthcare resources, thereby reinforcing the cyclical relationship between health and poverty. Additionally, the unique psychological and behavioral preferences of individuals in health poverty act as indirect factors that further strengthen this cycle. Health poverty individuals can generate endogenous force to escape the “health poverty trap” by enhancing their confidence levels and digital literacy. Therefore, this research proposes the following recommendations:

Grassroots civil affairs departments and communities should strive to accurately identify individuals experiencing health poverty, enhance the health status of their family members, and mitigate social health inequality. To achieve this, regions can establish identification systems during the health poverty identification phase, utilizing residents’ health records, family information records, and other pertinent data. In the post-health poverty alleviation era, poverty alleviation goals should not be limited to avoiding falling back into poverty due to illness or being impoverished by illness. Instead, the focus should be on improving the health status of socially disadvantaged groups and reducing overall health inequality.

Policymakers need to pay more attention to health inequality and enhance the cost-effectiveness of health interventions. Due to differences in policy objectives, if policymakers aim to develop more efficient strategies for health improvement, they should consider the psychological and behavioral characteristics of health poverty individuals. By reducing the workload and economic investment required in the actual intervention process, the cost-effectiveness of health interventions can be improved, and inequalities in psychological and functional health can be reduced.

From a national perspective, the Chinese government is steadfastly following the path of socialism and is committed to improving the health status of impoverished families. This commitment aligns with the shared goals of achieving a “Healthy China” and promoting “common prosperity”. To effectively intervene in the health of the impoverished population, it is essential to intensify efforts in disseminating health knowledge and enhancing public health literacy among the general population. This approach will empower individuals with the necessary information and skills to make informed decisions about their health, leading to improved health outcomes and reduced health disparities. Additionally, there is a need to enhance the provision of public fitness facilities and infrastructure in impoverished regions. This requires an increase in the allocation of human and material resources to the healthcare system. It is imperative to strengthen healthcare insurance and medical assistance programs, with a particular emphasis on improving the quality of medical services. Priority should be given to delivering high-quality primary healthcare resources to impoverished areas, aiming to reduce the gap of health inequality. By implementing these measures, it is possible to promote better health outcomes and address the healthcare needs of the impoverished population effectively.

Further research opportunities exist in this field. Future studies can concentrate on specific health policies or multidimensional poverty policies. Additionally, the next phase of this research aims to develop a predictive model that investigates the influence of poverty dimensions on individual health and forecasts the ramifications of alleviating specific poverty dimensions on individual health behavior variables. This approach will facilitate the formulation of more precise policy recommendations.

## Data Availability

Publicly available datasets were analyzed in this study. The data can be found here: http://isss.pku.edu.cn/cfps/download/index#/fileTreeList.

## Abbreviations

CFPS: China family panel studies
UQR: Unconditional quantile regression
CQR: Conditional quantile regression
RIF: Recentered influence function
SRH: Self-rated health
2SLS: Two-stage least squares
